# Work-related musculoskeletal disorders among facial plastic surgeons and rhinologists

**DOI:** 10.3389/fpubh.2026.1850408

**Published:** 2026-06-29

**Authors:** Feras Alkholaiwi, Ji Yun Choi

**Affiliations:** 1 Department of Otorhinolaryngology, Head and Neck Surgery, College of Medicine, Imam Mohammad Ibn Saud Islamic University (IMSIU), Riyadh, Saudi Arabia; 2 Department of Otorhinolaryngology, Head and Neck Surgery, Chosun University College of Medicine, Gwangju, Republic of Korea

**Keywords:** facial plastic surgeon, Nordic Musculoskeletal Questionnaire (NMQ), otolaryngologists, rhinologists, work-related musculoskeletal disorders (WMSDs)

## Abstract

**Background:**

Work-related musculoskeletal disorders (WMSDs) are a prevalent issue among facial plastic surgeons and rhinologists, largely due to the physically demanding and intricate nature of their work. These professionals often face prolonged periods of awkward postures, repetitive movements, and the need for precise, sustained focus, all of which contribute to the development of musculoskeletal problems over time. The frequent need for prolonged periods of precise movements and static postures during surgeries increases the risk of developing musculoskeletal issues.

**Objective:**

The study aimed to assess the frequency of work-related musculoskeletal disorders (WMSDs) among facial plastic surgeons and rhinologists. The study identified the key risk factors contributing to the development of these disorders and gained insight into how WMSDs affect the daily practice and overall performance of these surgical professionals.

**Methodology:**

This cross-sectional survey-based study was done in a 6-month period, from June to November 2023. The research utilized a specially designed questionnaire, Nordic Musculoskeletal Questionnaire (NMQ). This tailored survey allowed for a comprehensive evaluation of musculoskeletal health, targeting the specific challenges faced by facial plastic surgeons and rhinologists in their daily practice. After getting informed consent, a questionnaire was distributed to 54 rhinologists and facial plastic surgeons during a scientific meeting.

**Results:**

The study included a total of 54 surgeons, with an average age of 47.4 years (± 7.95). The majority of participants were male (79.6%), while female surgeons made up 20.4% of the sample. Notably, 72.2% of the respondents reported spending over 10 h per week in the operating room, with 33.0% logging more than 15 h of surgical time weekly. Work-related musculoskeletal disorders (WMSDs) were reported by 90.7% of otolaryngologists surveyed. The most common activity restriction was due to lower back (16.7%), neck (16.7%), shoulders (14.8%), and wrist/hand difficulties (11.1%). The most frequent cause for visiting a physician (13%) was lower back problems; the least common reasons were knees (3.7%) and wrists/hands (3.7%). Gender differences were noted, with a higher percentage of men reporting activity restrictions due to musculoskeletal issues (87.2% vs. 12.8%, *p* = 0.05).

**Conclusion:**

Facial plastic surgeons and rhinologists are frequently affected by WMSDs, particularly affecting the neck and the shoulder. Notably, our study revealed a concerning disconnect between ergonomic awareness and practice: while 25.9% of surgeons were aware of ergonomic principles, only 13% implemented them. This substantial gap underscores an urgent need for comprehensive ergonomic training programs and systematic implementation of ergonomic practices in otolaryngology training curricula and surgical departments.

## Introduction

According to the definition, “musculoskeletal disorders to which the work environment and the performance of work contribute significantly” are known as work-related musculoskeletal disorders (WMSDs) (1). These disorders arise primarily due to repetitive movements, awkward postures, or prolonged periods of strain that are often required in specific professions. Over time, such work-related activities can lead to discomfort, pain, or even chronic musculoskeletal conditions, highlighting the critical impact that workplace factors have on the health and wellbeing of individuals in these roles. Surgeons are highly prone to WMSDs due to the prolonged, awkward positions they must maintain during surgeries. These physically demanding tasks put significant strain on their bodies over time (2, 3).

Otolaryngologists, with different subspecialities like facial plastic surgery and rhinology face significant ergonomic risks that have not been thoroughly examined. The outpatient examination and surgical procedures require extended periods of sitting, contributing to a sedentary work environment. The work profile of an otolaryngologist involves performing keyhole procedures and performing examination of deeply situated, small cavities in outpatient settings. There is a restricted range of views and limited mobility. When using endoscopes, otoscopes, or microscopes and managing various equipment, otorhinolaryngologists maintain their neck, back, or shoulders in forced positions. This indicates that there is a chance of musculoskeletal problems (4, 5).

Surgical fatigue syndrome, which can result from musculoskeletal complaints, can impair a surgeon’s dexterity, judgment, and performance, which can be harmful to the patient as well as the surgeon (6). The growing number of publications on this issues, in the recent years suggests that otolaryngology is not exempt from the widespread concern regarding musculoskeletal disorders stemming from surgical practice across various specialties. Studies focusing on ENT specialists and those with specialized expertise have reported a relatively high incidence of musculoskeletal symptoms, which participants often associate with their surgical work (4–6). Implementing early training is regarded as the most effective way to raise awareness and provide essential knowledge on proper workplace ergonomics, positively influencing a surgeon’s career trajectory (7, 8). This study’s goal is to assess the prevalence of WMSD, identify risk factors, and evaluate its impact on surgical practice. The secondary objectives are to study the long-term impact of WMSD and the awareness about it.

## Materials and methods

This cross-sectional survey-based study was conducted from June to November 2023. During a scientific meetings in South Korea, a research team distributed the questionnaire to facial plastic surgeons and rhinologists. To qualify for inclusion in the study, participants needed to be board-certified with fellowship trained in facial plastic surgery or rhinology, actively engaged in their practice, and not retired. Non-facial plastic surgeons, or rhinologists, and not practicing surgeries were excluded from the study. This study was approved by the institutional review board, and informed consent was obtained from each respondent before survey initiation. Confidentiality was guaranteed to all respondents.

Informed consent was obtained from all participants before they were included in the study. Out of the 70 surgeons invited to participate, 54 responded to the questionnaire. The survey was based on the validated Nordic Musculoskeletal Questionnaire (NMQ) (9). It was divided into two sections. The first section collected demographic and professional data, such as gender, age, height, weight, years of postgraduate experience, average weekly hours spent in the operating room, dominant hand, average hours of sleep per night, and weekly exercise habits. The second section assessed the participants’ operating positions using a five-point Likert scale, where 1 represented “sitting all the time” and 5 represented “standing all the time.”

The second part of the questionnaire was designed to gather information about musculoskeletal symptoms experienced by participants in nine different areas of the body: the neck, shoulders, elbows, wrists/hands, upper back, lower back, hips/thighs, knees, and ankles/feet. This section followed the framework of the Nordic Musculoskeletal Questionnaire (NMQ). Participants were asked whether they had experienced any long-term issues (within the 12 months leading up to the survey) or recent symptoms (within the last 7 days) in these areas. In addition, the questionnaire inquired if the participants had sought medical advice for these problems and whether their daily activities had been disrupted over the past year due to these musculoskeletal issues. This approach provided a thorough understanding of the prevalence, severity, and impact of musculoskeletal complaints among the surgeons.

### Statistical analysis

Statistical analysis for the study was carried out using two software programs: the Statistical Package for Social Sciences (SPSS), Version 24.0, developed by IBM Corp. in Armonk, New York, USA, and RStudio, Version 1.3, from RStudio, Inc. in Boston, Massachusetts, USA. The analysis of the data was divided into two categories. For categorical data such as gender and age, the results were presented in the form of frequencies and percentages. For continuous data, were presented as means and standard deviation.

Additionally, the prevalence of complaints per anatomical region was reported as a frequency and percentage. Bar plots were also used to visualize the results. A chi-square test was conducted to assess whether age, gender, operating position, or the average number of operating hours were significantly associated with musculoskeletal complaints in any of the nine anatomic regions. The level of statistical significance was set at 0.05.

## Results

The study sample included 54 respondents, including 43 (79.6%) males and 11 (20.4%) females. The average age of the participants was 47.4 ± 7.95 years. Most respondents were involved in teaching residents or fellows during operations (85.2%). Most respondents were working in university hospitals (74.1%), and 40.7% had been practicing for 10–20 years post-residency. More than one-third of the respondents were overweight (40.7%). Most of the respondents were practicing both rhinology and facial plastic surgery (74.1%) (Table 1).

**Table 1:** Descriptive statistics for the study sample.

Parameters	Total
Age	47.4 ± 7.95
Gender	
Female	11 (20.4%)
Male	43 (79.6%)
Marital status	
Married	49 (90.7%)
Unmarried	5 (9.26%)
Are you a smoker?	5 (9.26%)
Average hours of sleep per day	6.46 ± 0.77
Participate in teaching residents or fellows during operations.	46 (85.2%)
Medical institution	
Private hospital/clinic	14 (25.9%)
University Hospital	40 (74.1%)
Practice	
Both rhinology and facial plastic surgery	40 (74.1%)
Facial plastic surgery only	2 (3.70%)
Rhinology only	12 (22.2%)
Practice years after residency	
< 10 years	13 (24.1%)
10–20 years	22 (40.7%)
≥ 20 years	19 (35.2%)
Weight (in kg)	71.7 ± 12.2
Height (in cm)	172 ± 7.67
BMI (Kg/m^2^)	24.1 ± 2.90
BMI category	
Normal	32 (59.3%)
Overweight	22 (40.7%)

Regarding risk factors, approximately two-thirds of the respondents reported spending more than 10 h in the operating room per week (72.2%), with 33.3% spending more than 15 h. Half of the respondents reported standing during operations most of the time (48.1%), while 24.1% stood throughout the entire time. Half of the respondents reported experiencing it regularly (53.7%), while 42.6% reported performing long surgeries at least once a week. One-third of the respondents had suffered sports injuries (35.2%). A similar number previously had undergone physical therapy due to work-related MS complaints (33.3%). Only 25.9% of the respondents were aware of the surgical ergonomics recommendations, with just 13% applying this knowledge in practice. A larger proportion (61.6%) of the respondents were aware of obstacles facing surgeons regarding work-related MS symptoms (Table 2).

**Table 2:** Risk factors for MS problems (*N =* 54).

Risk factors	Number (%)
Number of hours in the operating room per week	
<10 h	15 (27.8%)
10–15 h	21 (38.9%)
≥15 h	18 (33.3%)
Operating position	
a. Sitting most of the time	2 (3.70%)
b. Sometimes sitting/Sometimes standing	13 (24.1%)
c. Standing most of the time	26 (48.1%)
d. Standing all the time	13 (24.1%)
Exercise regularly	
No	25 (46.3%)
Yes	29 (53.7%)
Hours of exercise per week	
I do not exercise	4 (7.41%)
<2 h/week	18 (33.3%)
2–4 h/week	19 (35.2%)
5–7 h/week	7 (13.0%)
More than 7 h/week	6 (11.1%)
Practice long surgeries at least once a week (≥ 4 consecutive hours)	23 (42.6%)
Ever suffered any sports injuries?	19 (35.2%)
Ever done any physical therapy (due to work-related MS complaints)?	18 (33.3%)
Aware of the recommendations made by the field of surgical ergonomics	14 (25.9%)
Ever applied any of this information to surgical practice?	7 (13.0%)
Aware of surgeons facing obstacles regarding work-related MS symptoms	33 (61.1%)

**Figure 1:**
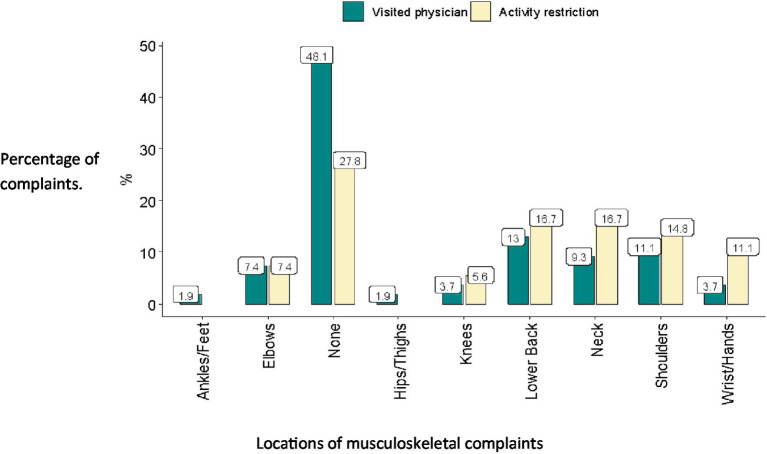
Impact of musculoskeletal complaints (% is reported based in the entire sample).

More than half of the respondents visited the physician within the past year due to their MS complaints (51.9%). Activity restrictions due to MS problems in the past 12 months were reported by 72.2% of the respondents (Table 3). The main reasons for activity restriction were problems with the lower back (16.7%), neck (16.7%), shoulders (14.8%), and wrists/hands (11.1%). Lower back problems were the highest reason for seeing the physician (13%) while knees (3.7%) and wrists/hands (3.7%) were the least common reasons (Figure 1).

**Table 3:** Factors associated with visiting the physician and activity restriction over the past year.

Factors associated with:	Activity restriction		*P*	Visiting physician		*P*
	No	Yes		No	Yes	
	*N =* 15	*N =* 39		*N =* 26	*N =* 28	
Age	46.1 (10.4)	47.9 (6.87)	0.545	45.9 (8.65)	48.8 (7.10)	0.187
Gender			0.05			0.890
Female	6 (40.0%)	5 (12.8%)		6 (23.1%)	5 (17.9%)	
Male	9 (60.0%)	34 (87.2%)		20 (76.9%)	23 (82.1%)	
Marital status			1.000			1.000
Married	14 (93.3%)	35 (89.7%)		24 (92.3%)	25 (89.3%)	
Unmarried	1 (6.67%)	4 (10.3%)		2 (7.69%)	3 (10.7%)	
Weight (in kg)	64.3 (12.7)	74.6 (10.8)	0.011	70.0 (13.5)	73.4 (10.9)	0.321
Height (in cm)	169 (9.95)	173 (6.35)	0.135	172 (8.82)	172 (6.59)	0.849
Are you a smoker?	0 (0.00%)	5 (12.8%)	0.306	3 (11.5%)	2 (7.14%)	0.663
Average hours of sleep per day	6.53 (1.06)	6.44 (0.64)	0.743	6.54 (0.86)	6.39 (0.69)	0.497
Participate in teaching residents or fellows during operations	12 (80.0%)	34 (87.2%)	0.671	22 (84.6%)	24 (85.7%)	1.000
Medical institution			0.318			0.454
Private hospital/clinic	5 (33.0%)	9 (23.0%)		5 (19.2%)	9 (36.0%)	
University Hospital	10 (66.7%)	30 (76.9%)		21 (80.8%)	19 (67.9%)	
Practice years after residency			0.737			0.788
<10 years	4 (26.7%)	9 (23.1%)		7 (26.9%)	6 (21.4%)	
10–20 years	7 (46.7%)	15 (38.5%)		11 (42.3%)	11 (39.3%)	
≥20 years	4 (26.7%)	15 (38.5%)		8 (30.8%)	11 (39.3%)	
Number of hours in the operating room per week			0.036			0.233
<10 h	8 (53.3%)	7 (17.9%)		10 (38.5%)	5 (17.9%)	
10–15 h	3 (20.0%)	18 (46.2%)		9 (34.6%)	12 (42.9%)	
≥15 h	4 (26.7%)	14 (35.9%)		7 (26.9%)	11 (39.3%)	
Practice long surgeries at least once a week (≥4 consecutive hours)	6 (40.0%)	17 (43.6%)	1.000	9 (34.6%)	14 (50.0%)	0.386
Practice type			0.608			0.590
Both rhinology and facial plastic	10 (66.7%)	30 (76.9%)		20 (76.9%)	20 (71.4%)	
Facial plastic surgery only	1 (6.67%)	1 (2.56%)		0 (0.00%)	2 (7.14%)	
Rhinology only	4 (26.7%)	8 (20.5%)		6 (23.1%)	6 (21.4%)	
Operating position			0.387			0.527
Sitting most of the time	0 (0.00%)	2 (5.13%)		0 (0.00%)	2 (7.14%)	
Sometimes sitting/Sometimes standing	2 (13.3%)	11 (28.2%)		8 (30.8%)	5 (17.9%)	
Standing most of the time	10 (66.7%)	16 (41.0%)		12 (46.2%)	14 (50.0%)	
Standing all the time	3 (20.0%)	10 (25.6%)		6 (23.1%)	7 (25.0%)	
Exercise regularly	9 (60.0%)	20 (51.3%)	0.787	15 (57.7%)	14 (50.0%)	0.769
Ever suffered any sports injuries?	3 (20.0%)	16 (41.0%)	0.258	6 (23.1%)	13 (46.4%)	0.131
Have you done any physical therapy (due to work-related musculoskeletal complaints)?	3 (20.0%)	15 (38.5%)	0.334	2 (7.69%)	16 (57.1%)	<0.001
Are you aware of the recommendations made by the field of surgical ergonomics, its studies, and its research?	3 (20.0%)	11 (28.2%)	0.733	5 (19.2%)	9 (32.1%)	0.441
Applied any of this information to practice	2 (13.3%)	5 (12.8%)	1.000	3 (11.5%)	4 (14.3%)	1.000
Aware of the obstacles faced by surgeons regarding work-related MD symptoms?	8 (53.3%)	25 (64.1%)	0.678	14 (53.8%)	19 (67.9%)	0.438
BMI	22.3 (2.60)	24.8 (2.74)	0.005	23.5 (2.84)	24.7 (2.89)	0.145
BMI category			0.026			0.246
Normal	13 (86.7%)	19 (48.7%)		18 (69.2%)	14 (50.0%)	
Overweight	2 (13.3%)	20 (51.3%)		8 (30.8%)	14 (50.0%)	

The analysis involved the Chi-square test of independence for categorical data, while continuous data were compared using an unpaired *t*-test. Categorical variables were summarized as counts and percentages, and continuous variables were reported as mean (standard deviation).

**Figure 2:**
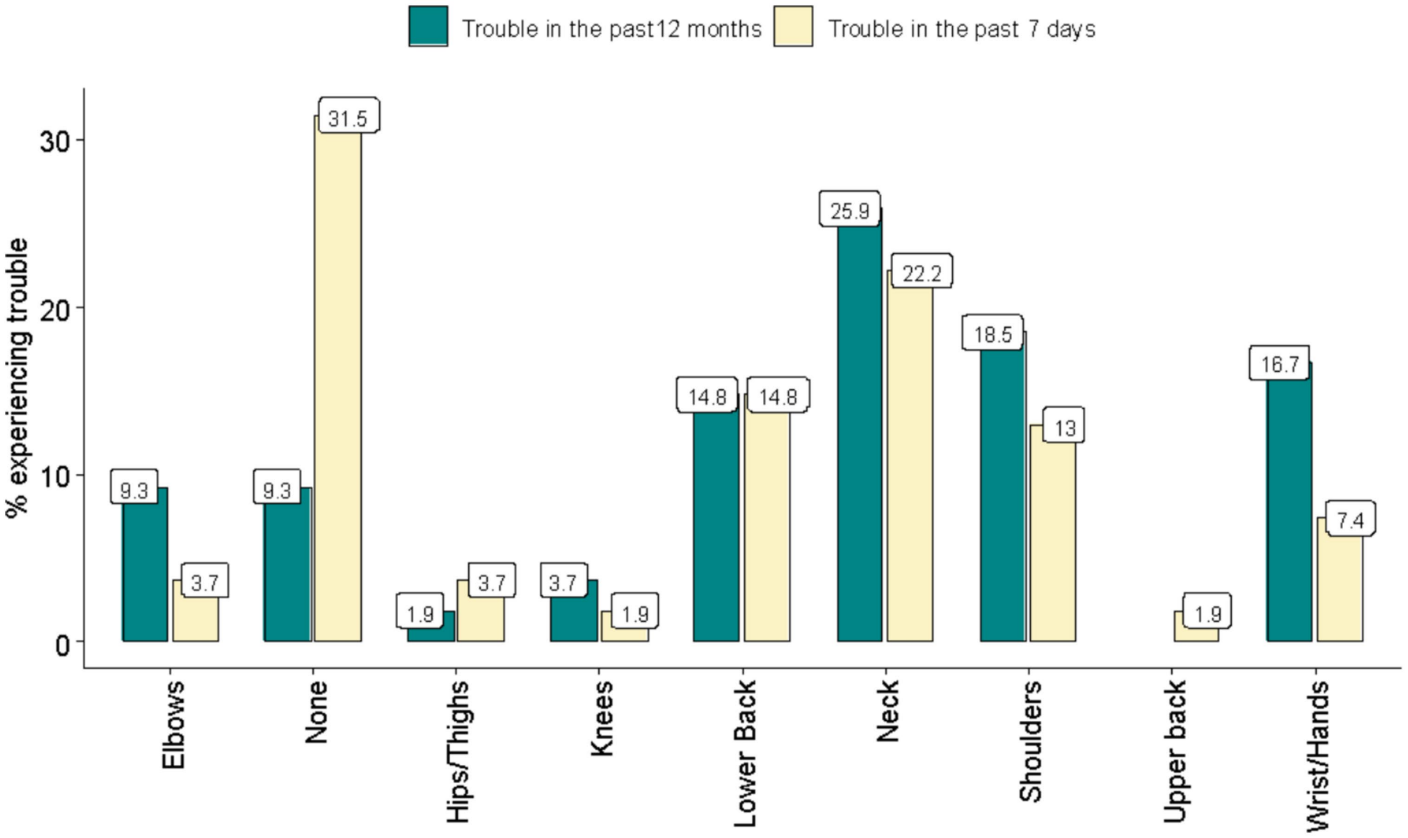
Musculoskeletal complaints stratified by time (% are reported in white boxes).

The most common sites for MS problems were neck (~25%), lower back (14.8%), shoulders (13, 18.5% over the past 7 days and 12 months, respectively), and wrists/hands (7.4 and 16.7%, respectively) (Figure 2).

**Figure 3:**
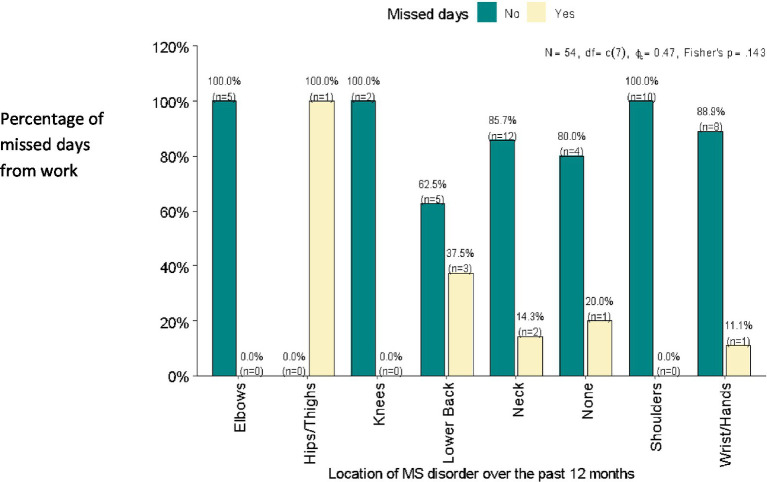
Association between the reason for activity restriction and missed days from work.

Eight respondents (14.8%) reported missing days from work over the past year due to MS problems. Although not statistically significant, respondents with MS problems in hips/thighs, wrists/hands, and lower back were more likely to miss days from work (Figure 3).

Male gender was significantly associated with activity restriction due to MS problems (*p* = 0.05), with a higher percentage of males reporting activity restriction than females due to MS problems (87.2% vs. 12.8%). Additionally, higher weight and BMI were associated with a higher probability of activity restriction due to MS problems. More hours spent in the operating room also showed a significant association with activity restriction due to MS problems (*p* = 0.036).

None of the other factors were significantly associated with activity restriction or physician visits for MS problems. However, respondents who had visited a physician in the past 12 months were most likely to require physical therapy (*p* < 0.001) (Table 3).

Only one-third of the respondents reported being free of MS problems over the past week, in contrast to just 9.3% over the past years. A higher portion of males (81.6%) reported MS problems compared to females (18.4%), but this difference was not statistically significant (*p* = 0.266). Factors such as duration of surgeries, operating position, practice type, exercise, and sports injury did not show any significant relationship with MS problems (Table 4).

**Table 4:** Demographic characteristics associated with MS problems over the past 12 months.

Demographic characteristics	No problem	MS problem	*P*
	*N =* 5	*N =* 49	
Gender			0.266
Female	2 (40.0%)	9 (18.4%)	
Male	3 (60.0%)	40 (81.6%)	
Age	48.2 (12.9)	47.3 (7.48)	0.891
Number of hours in the operating room per week?			0.356
[0, 10)	3 (60.0%)	12 (24.5%)	
[10, 15)	1 (20.0%)	20 (40.8%)	
[15, 100]	1 (20.0%)	17 (34.7%)	
Do you practice long surgeries (≥ 4 consecutive hours) at least once a week?			1.000
No	3 (60.0%)	28 (57.1%)	
Yes	2 (40.0%)	21 (42.9%)	
Mainly, my practice is			0.445
Both rhinology and facial plastic	3 (60.0%)	37 (75.5%)	
Facial plastic surgery only	0 (0.00%)	2 (4.08%)	
Rhinology only	2 (40.0%)	10 (20.4%)	
Operating position			1.000
Sitting most of the time	0 (0.00%)	2 (4.08%)	
Sometimes sitting/Sometimes standing	1 (20.0%)	12 (24.5%)	
Standing most of the time	3 (60.0%)	23 (46.9%)	
Standing all the time	1 (20.0%)	12 (24.5%)	
Do you exercise regularly?			1.000
No	2 (40.0%)	23 (46.9%)	
Yes	3 (60.0%)	26 (53.1%)	
How many hours do you exercise per week? (h/week)			0.761
a. I do not exercise	0 (0.00%)	4 (8.16%)	
b. < 2 h/week	2 (40.0%)	16 (32.7%)	
c. 2–4 h/week	1 (20.0%)	18 (36.7%)	
d. 5–7 h/week	1 (20.0%)	6 (12.2%)	
e. more than 7 h/week	1 (20.0%)	5 (10.2%)	
Have you suffered any sports injuries?			0.646
No	4 (80.0%)	31 (63.3%)	
Yes	1 (20.0%)	18 (36.7%)	
Have you done any physical therapy (due to work-related MS complaints)?			0.655
No	4 (80.0%)	32 (65.3%)	
Yes	1 (20.0%)	17 (34.7%)	
Aware of the recommendations made by the field of surgical ergonomics			0.595
No	3 (60.0%)	37 (75.5%)	
Yes	2 (40.0%)	12 (24.5%)	
Have you applied any of this information to your surgical practice?			0.120
No	3 (60.0%)	44 (89.8%)	
Yes	2 (40.0%)	5 (10.2%)	
Aware of the obstacles faced by surgeons regarding work-related MS symptoms			1.000
No	2 (40.0%)	19 (38.8%)	
Yes	3 (60.0%)	30 (61.2%)	

Data were summarized using counts and percentages. Analysis was performed using the Chi-square test of independence.

## Discussion

Work-related musculoskeletal disorders (WMSDs) are common among otolaryngologists, with prior studies reporting prevalence rates of 62–83% across specific subgroups of this profession (1, 5). In this study, an overwhelming majority of facial plastic surgeons and rhinologists (90.7%) were found to suffer from work-related musculoskeletal disorders (WMSDs). This finding is consistent with the results of a Canadian study, which reported an even higher rate of 97% among otolaryngologists (10). Similarly, Vijendran et al. (9) observed that 47.4% of otorhinolaryngologists in their study experienced musculoskeletal issues. Meanwhile, Cavanagh et al. (11) reported a 62% incidence of these problems specifically among pediatric otorhinolaryngologists. These figures highlight the widespread nature of WMSDs within the field of otolaryngology, affecting a significant portion of specialists across different regions and subspecialties.

Our study included 54 participants with a mean age of 47.4 years. The participant’s mean weight (71.7 ± 12.2 kg), height (172 ± 7.67 cm), and BMI (24.1 ± 2.90 kg/m^2^) in our study were identical to those reported by Dahmash et al. (12) study, where participants had a mean weight of 73.69 ± 15.20 kg, mean heights of 170.78 ± 8.13 cm, and mean BMIs of 25.10 ± 3.77 kg/m^2^ SD. The average sleep duration (6.46 ± 0.77 h) also mirrored that of the Dahmash et al. (12) study. However, while Dahmash et al. (12) reported that participants spent 35.6% of their time standing during surgeries, 48.1% of our respondents reported standing most of the time during procedures.

In a similar vein, research conducted by Bolduc-Bégin et al. (10) found that the majority of surgeons spent between 8 and 12 h per week in the operating room. This aligns closely with the findings of our study, where 38.9% of respondents reported spending between 10 and 15 h per week performing surgeries. We also observed a positive correlation between longer operating hours and activity restrictions due to musculoskeletal issues (*p* = 0.036), further underscoring the impact of extended surgical hours on surgeon health.

In our study, the primary causes of the activity restriction were lower back (16.7%), neck (16.7%), and shoulder pain (14.8%), consistent with the findings of Dahmash et al. (12), who identified that lower back symptoms (24.4%) and shoulder issues (13.3%) were the most frequent reasons for activity limitation. Interestingly, in our study, male gender was significantly associated with higher rates of activity restriction related to MS problems (*p* = 0.05), with 87.2% of males reporting such restrictions, compared to only 12.8% of females. This finding contrasts with Wong et al. (1) research, where female surgeons more commonly experienced limitations due to knees (33.3%), elbows (27%), and hand/wrist (27%).

Mirmohammadi et al. (13) reported a similar observation in their study conducted in Iran, where they identified a notable difference in the prevalence of work-related musculoskeletal disorders (WMSDs) between female and male nurses. Previous research on musculoskeletal issues among female surgeons has found a higher frequency of symptoms in women, particularly in specific body regions (14, 15). A significant correlation was found between the female gender and musculoskeletal problems, which could be attributed to factors such as lower upper body strength and equipment design for larger male hands (16). Notably, no link was found between musculoskeletal problems and posture. The increasing proportion of female otolaryngologists raises the possibility that women are more likely to report these kinds of problems (17).

In our study, the most reported musculoskeletal (MS) complaints over the past 12 months were neck (25%), lower back (14.8%), shoulders (13%), and hands (16.7%). These findings align with Wong et al. (1), who reported neck stiffness (71.6%), neck pain (61.7%), and lower back pain (48.2%) as the most frequent symptoms. Another study found that men reported neck issues most often (63.6%), while women reported shoulder complaints (75%). This result is consistent with findings from other surgical specialties, including orthopedics (18), plastic surgery (19), oral surgery (20, 21), and maxillofacial surgery (20), indicating that the neck and back are most likely to be strained during any surgical procedure.

Facial plastic surgeons and rhinologists are prone to work-related musculoskeletal disorders, particularly in the neck, shoulders, and lower back. To mitigate these risks, ergonomics training is essential for improving both surgeons’ wellbeing and patient care. This study highlights the need to take ergonomics into account when estimating workload because it found that higher work hours were associated with a higher risk of musculoskeletal symptoms. Instructing trainees in surgical ergonomics may help lower the incidence and severity of musculoskeletal disorders in our area.

Prolonged standing during facial plastic and rhinological surgeries as in our study (71.4%) could increase the muscle loading in the neck, lower back, and lower extremities as well. Unlike seating position such as in different other otolaryngological procedures specially with using microscope and surgeons can maintain trunk position.

Early training for surgical staff to prevent musculoskeletal symptoms is essential, like proper adjustment of surgical table, monitors positions, adjustable surgical stools. In addition, micro-breaks during long procedures, stretching exercises between surgical cases, alternating between standing and seated positions if possible in lengthy surgeries. It is advisable to provide the training residents about preventions measures, with conducting ergonomic assessment, covering biomechanics of procedures postures, and knowing risk factors for work related musculoskeletal symptoms.

The findings of our study must be interpreted considering several limitations. Firstly, the small sample size is a limitation, though we achieved an excellent response rate of 77.14%, which compares favorably with similar studies in the literature. There is also a potential for selection bias as surgeons interested in ergonomics or having experienced musculoskeletal symptoms may have been more likely to participate in the survey. Additionally, the cross-sectional design limits the ability to draw causal inferences, and recall bias may have affected the accuracy of self-reported data.

Despite these limitations, our study provides valuable insights into the prevalence of musculoskeletal disorders among otolaryngology practitioners. Future studies should focus on evaluating interventions aimed at implementing ergonomic principles and equipment. Future longitudinal studies on WMSDs are recommended to understand the long-term effects better. It is also important to assess how specific ergonomic modifications during surgery could reduce the intensity and prevalence of WMSDs in this field.

## Conclusion

Facial plastic surgeons and rhinologists are frequently affected by WMSDs, particularly affecting the neck and the shoulder. Notably, our study revealed a concerning disconnect between ergonomic awareness and practice: while 25.9% of surgeons were aware of ergonomic principles, only 13% implemented them. This substantial gap underscores an urgent need for comprehensive ergonomic training programs and systematic implementation of ergonomic practices in otolaryngology training curricula and surgical departments.

## Data Availability

The original contributions presented in the study are included in the article/supplementary material, further inquiries can be directed to the corresponding author.
